# Gene model for the ortholog of *Glys* in *Drosophila yakuba*

**DOI:** 10.17912/micropub.biology.000983

**Published:** 2024-12-18

**Authors:** Alyssa C. Koehler, Logan Cohen, Isaac Romo, Viet Le, James J. Youngblom, Amy T. Hark, Chinmay P. Rele, Laura K Reed

**Affiliations:** 1 University of Alabama, Tuscaloosa, AL US; 2 Worcester State University, Worcester MA, USA; 3 California State University Stanislaus, Turlock, CA USA; 4 Muhlenberg College, Allentown, PA, USA

## Abstract

Gene model for the ortholog of *glycogen synthase* ( *Glys* ) in the May 2011 (WUGSC dyak_caf1/DyakCAF1) Genome Assembly (GenBank Accession: GCA_000005975.1 ) of *Drosophila yakuba* . This ortholog was characterized as part of a developing dataset to study the evolution of the Insulin/insulin-like growth factor signaling pathway (IIS) across the genus *Drosophila* using the Genomics Education Partnership gene annotation protocol for Course-based Undergraduate Research Experiences.

**Figure 1. Glys ortholog gene model comparison between D. yakuba and D. melanogaster:**
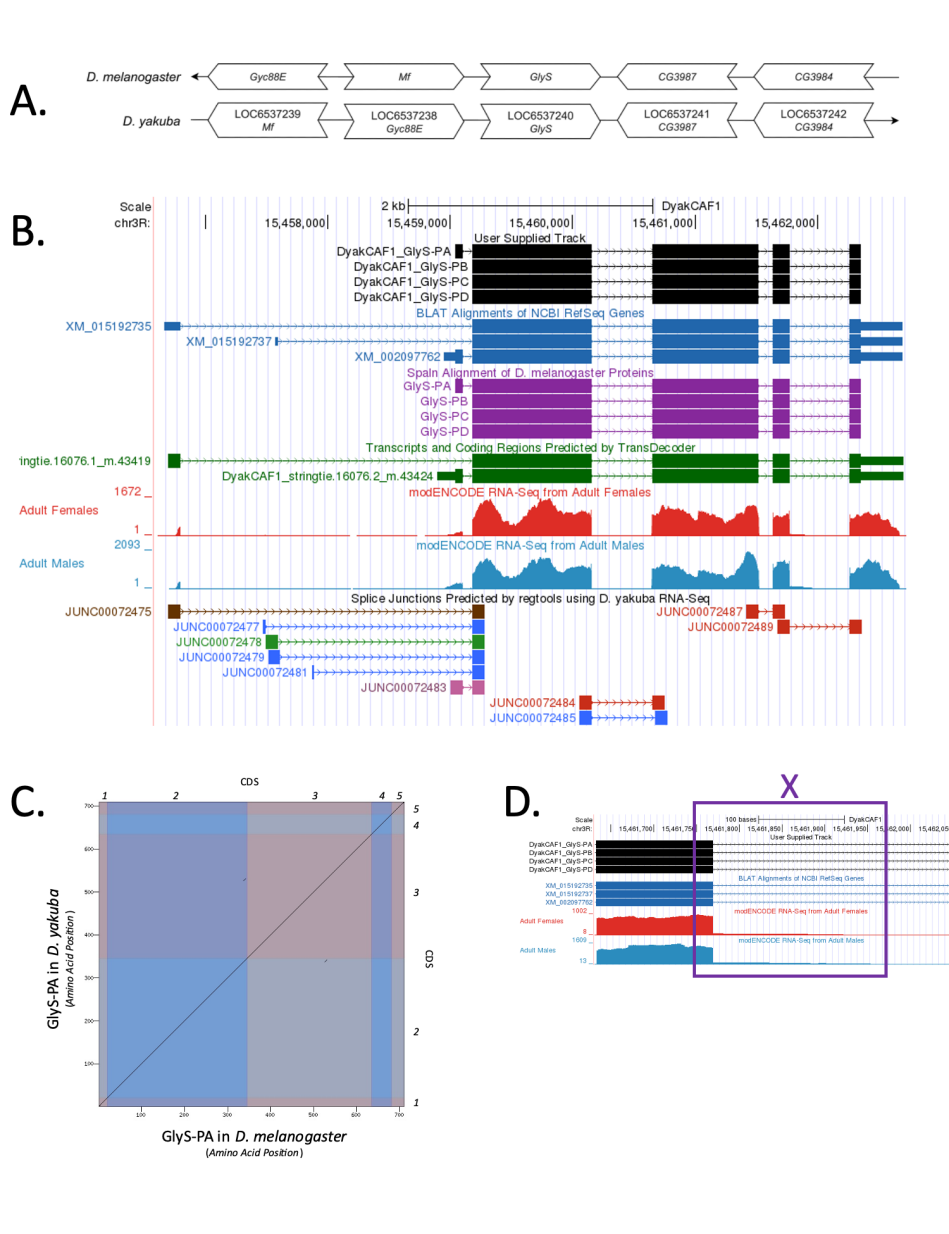
**
(A) Synteny comparison of the genomic neighborhoods for *Glys* in *Drosophila melanogaster* and *D. yakuba* .** Thin underlying arrows indicate the DNA strand within which the target gene– *Glys* –is located in *D. melanogaster* (top) and *D. yakuba* (bottom). The thin arrow pointing to the right indicates that *Glys* is on the positive (+) strand in *D. yakuba* , and the thin arrow pointing to the left indicates that *Glys* is on the negative (-) strand in *D. melanogaster* . The wide gene arrows pointing in the same direction as *Glys* are on the same strand relative to the thin underlying arrows, while wide gene arrows pointing in the opposite direction of *Glys* are on the opposite strand relative to the thin underlying arrows.White gene arrows in *D. yakuba* indicate orthology to the corresponding gene in *D. melanogaster* . Gene symbols given in the *D. yakuba* gene arrows indicate the orthologous gene in *D. melanogaster* , while the locus identifiers are specific to *D. yakuba* . **(B) Gene Model in GEP UCSC Track Data Hub (Raney et al., 2014).** The coding-regions of *Glys* in *D. yakuba* are displayed in the User Supplied Track (black); CDS are depicted by thick rectangles and introns by thin lines with arrows indicating the direction of transcription. Subsequent evidence tracks include BLAT Alignments of NCBI RefSeq Genes (dark blue, alignment of Ref-Seq genes for *D. yakuba* ), Spaln of *D. melanogaster* Proteins (purple, alignment of Ref-Seq proteins from *D. melanogaster* ), Transcripts and Coding Regions Predicted by TransDecoder (dark green), RNA-Seq from Adult Females and Adult Males (red and light blue, respectively; alignment of Illumina RNA-Seq reads from *D. yakuba* ), and Splice Junctions Predicted by regtools using *D. yakuba* RNA-Seq ( SRP006203 ). Splice junctions shown have a read-depth of 13-26, 60, 387, 810 and 1796-2030 supporting reads in blue, green, pink, brown, and red, respectively. **(C) Dot Plot of Glys-PA in *D. melanogaster* ( *x* -axis) vs. the orthologous peptide in *D. yakuba* ( *y* -axis).** Amino acid number is indicated along the left and bottom; CDS number is indicated along the top and right. CDS are also highlighted with alternating colors. **(D) UCSC Genome Browser displaying intronic RNA-Seq coverage within intron four of Glys-RA and intron three of Glys-RB, Glys-RC, and Glys-RD in *D. yakuba* .** The coding-regions of *Glys* in *D. yakuba* are displayed in the User Supplied Track (black); CDS are depicted by thick rectangles and introns by thin lines with arrows indicating the direction of transcription. Subsequent evidence tracks include BLAT Alignments of NCBI RefSeq Genes (dark blue, alignment of Ref-Seq genes for *D. yakuba* ) and RNA-Seq from Adult Females and Adult Males (red and light blue, respectively; alignment of Illumina RNA-Seq reads from *D. yakuba* ). The purple box denoted X highlights the higher coverage than expected in intron four for *Glys-RA* and intron three for *Glys-RB, Glys-RC* , and *Glys-RD* .

## Description

**Table:** 

*This article reports a predicted gene model generated by undergraduate work using a structured gene model annotation protocol defined by the Genomics Education Partnership (GEP; thegep.org) for Course-based Undergraduate Research Experience (CURE). The following information in this box may be repeated in other articles submitted by participants using the same GEP CURE protocol for annotating Drosophila species orthologs of Drosophila melanogaster genes in the insulin signaling pathway.* "In this GEP CURE protocol students use web-based tools to manually annotate genes in non-model *Drosophila* species based on orthology to genes in the well-annotated model organism fruitfly *Drosophila melanogaster* . The GEP uses web-based tools to allow undergraduates to participate in course-based research by generating manual annotations of genes in non-model species (Rele et al., 2023) . Computational-based gene predictions in any organism are often improved by careful manual annotation and curation, allowing for more accurate analyses of gene and genome evolution (Mudge and Harrow 2016; Tello-Ruiz et al., 2019) . These models of orthologous genes across species, such as the one presented here, then provide a reliable basis for further evolutionary genomic analyses when made available to the scientific community.” (Myers et al., 2024) . “The particular gene ortholog described here was characterized as part of a developing dataset to study the evolution of the Insulin/insulin-like growth factor signaling pathway (IIS) across the genus *Drosophila* . The Insulin/insulin-like growth factor signaling pathway (IIS) is a highly conserved signaling pathway in animals and is central to mediating organismal responses to nutrients (Hietakangas and Cohen 2009; Grewal 2009) .” (Myers et al., 2024) . “ *Glycogen synthase* ( *Glys* ; aka. *GS, GlyS* ) is a gene within the Insulin-signaling pathway in *Drosophila* and encodes a glycosyltransferase that catalyzes linkage of glucose monomers into glycogen. Glys activity is regulated allosterically by glucose 6-phosphate and phosphorylation/dephosphorylation allowing for control of cellular glycogen levels (Plyte et al., 1992; Roach et al., 2012) . Null *Glys* mutants exhibit growth defects and reduced larval viability in *Drosophila* (Yamada et al., 2019) .” (Backlund et al., 2024) .

We propose a gene model for the *D. yakuba* ortholog of the *D. melanogaster* *Glycogen synthase* ( *Glys* ) gene, the summary of which can be found in the box above. The genomic region of the ortholog corresponds to the uncharacterized protein XP_002097798.1 (Locus ID LOC6537240 ) in the Dyak_CAF1 Genome Assembly of *D. yakuba* (GenBank Accession: GCA_000005975.1 - Graveley et al., 2011). This model is based on RNA-Seq data from *D. yakuba* ( SRP006203 ) and *Glys* in *D. melanogaster* using FlyBase release FB2022_04 ( GCA_000001215.4 ; Larkin et al *.* , 2021).

*D. yakuba (* NCBI:txid7245) is part of the *melanogaster* species group within the subgenus *Sophophora* of the genus *Drosophila* (Sturtevant 1939; Bock and Wheeler 1972) . It was first described by Burla (1954). *D. yakuba* is wide-spread in sub-Saharan Africa and Madagascar (Lemeunier et al., 1986;  https://www.taxodros.uzh.ch, accessed 1 Feb 2023; Markow and O'Grady 2005) where figs served as a primary host along with other rotting fruits (Lachaise and Tsacas 1983) .

***Synteny***

The target gene, *Glys* , occurs on chromosome 3R in *D. melanogaster* and is flanked upstream by *Guanylyl cyclase at 88E* ( *Gyc88E* ) and *Myofilin* ( *Mf* ) and downstream by *CG3987* and *CG3984* . The *tblastn* search of *D. melanogaster* Glys-PA (query) against the *D. yakuba* (GenBank Accession: GCA_000005975.1 ) Genome Assembly (database) placed the putative ortholog of *Glys* within scaffold chromosome 3R (CM000160.2) at locus LOC6537240 ( XP_002097798.1 )— with an E-value of 0.0 and a percent identity of 99.09%. Furthermore, the putative ortholog is flanked upstream by LOC6537238 ( XP_039232370.1 ) and LOC6537239 ( XP_015048219.1 ), which correspond to *Gyc88E* and *Mf* in *D. melanogaster* (E-value: 0.0 and 0.0; identity: 95.56% and 99.18%, respectively, as determined by *blastp* ; Figure 1A, A ltschul et al *.* , 1990). The putative ortholog of *Glys* is flanked downstream by LOC6537241 ( XP_002097799.1 ) and LOC6537242 ( XP_002097800.1 ), which correspond to *CG3987* and *CG3984* in *D. melanogaster* (E-value: 5e-157 and 4e-110; identity: 74.33% and 78.05%, respectively, as determined by *blastp* ). The putative ortholog assignment for *Glys* in *D. yakuba* is supported by the following evidence: The genes surrounding the *Glys* ortholog are orthologous to the genes at the same locus in *D. melanogaster* and local synteny is completely conserved, supported by results generated from *blastp* ; we conclude that LOC6537240 is the correct ortholog of *Glys* in *D. yakuba* ( Figure 1A ).

***Protein Model***

*Glys* in *D. yakuba* has two unique protein-coding isoforms Glys-PA and Glys-PC (identical to Glys-PB and Glys-PD; Figure 1B ). mRNA isoform (Glys-RA) contains five CDSs. mRNA isoforms Glys-RC, Glys-RB and Glys-RD, which differ in their 5' UTRs, contain four CDSs. Relative to the ortholog in *D. melanogaster* , the RNA CDS number and protein isoform count are conserved. The sequence of Glys-PA in *D. yakuba* has 100.00% identity (E-value: 0.0) with the protein-coding isoform Glys-PA in *D. melanogaster* , as determined by *blastp* and illustrated by dot plot in *Figure 1C* . There appears to be high RNA-Seq coverage in the fourth intron and third intron of Glys-PA and Glys-PB, displayed in Figure 1D . Coordinates of this curated gene model are stored by NCBI at GenBank/BankIt (accession BK064679 , BK064680 , BK064681 , BK064682 ). These data are also archived in the CaltechDATA repository (see “Extended Data” section below).

***Special characteristics of the protein model***

**Intronic RNA-Seq Coverage in intron four of *Glys-RA* and intron three of *Glys-RC* :** There is higher coverage than expected in intron four for *Glys-RA* and intron three for *Glys-RB, Glys-RC* , and *Glys-RD* , highlighted by the purple box denoted X ( Figure 1D ). One possible explanation is that RNA-Seq coverage could be from another location in the genome that has high sequence similarity, causing incorrect mapping of the RNA-Seq reads. To test this hypothesis, a *blastn* search was performed using the intronic DNA as the query sequence and the *D. yakuba* genome as the subject. This showed no results other than the *Glys* ortholog, suggesting the RNA-Seq coverage was mapped to the correct region. It is also possible that the high levels of intronic RNA-coverage could represent a novel isoform. High RNA-Seq coverage in this region has been observed in other species including *D. eugracilis* , *D. elegans* , and *D. ficusphila* . While this evidence supports a novel isoform, we do not have the data to definitively conclude the presence of a novel isoform in *D. yakuba* .

## Methods

Detailed methods including algorithms, database versions, and citations for the complete annotation process can be found in Rele et al. (2023). Briefly, students use the GEP instance of the UCSC Genome Browser v.435 (https://gander.wustl.edu ; Kent WJ et al., 2002; Navarro Gonzalez et al., 2021) to examine the genomic neighborhood of their reference IIS gene in the *D. melanogaster* genome assembly (Aug. 2014; BDGP Release 6 + ISO1 MT/dm6). Students then retrieve the protein sequence for the *D. melanogaster* target gene for a given isoform and run it using *tblastn* against their target *Drosophila* species genome assembly ( *Drosophila yakuba* ( GCA_000005975.1 )- Graveley et al., 2010)) on the NCBI BLAST server (https://blast.ncbi.nlm.nih.gov/Blast.cgi, Altschul et al., 1990) to identify potential orthologs. To validate the potential ortholog, students compare the local genomic neighborhood of their potential ortholog with the genomic neighborhood of their reference gene in *D. melanogaster* . This local synteny analysis includes at minimum the two upstream and downstream genes relative to their putative ortholog. They also explore other sets of genomic evidence using multiple alignment tracks in the Genome Browser, including BLAT alignments of RefSeq Genes, Spaln alignment of *D. melanogaster* proteins, multiple gene prediction tracks (e.g., GeMoMa, Geneid, Augustus), and modENCODE RNA-Seq from the target species. Genomic structure information (e.g., CDSs, CDS number and boundaries, number of isoforms) for the *D. melanogaster* reference gene is retrieved through the Gene Record Finder (https://gander.wustl.edu/~wilson/dmelgenerecord/index.html; Rele et al *.,* 2023). Approximate splice sites within the target gene are determined using *tblastn* using the CDSs from the *D. melanogaste* r reference gene. Coordinates of CDSs are then refined by examining aligned modENCODE RNA-Seq data, and by applying paradigms of molecular biology such as identifying canonical splice site sequences and ensuring the maintenance of an open reading frame across hypothesized splice sites. Students then confirm the biological validity of their target gene model using the Gene Model Checker (https://gander.wustl.edu/~wilson/dmelgenerecord/index.html; Rele et al., 2023), which compares the structure and translated sequence from their hypothesized target gene model against the *D. melanogaster* reference gene model. At least two independent models for each gene are generated by students under mentorship of their faculty course instructors. These models are then reconciled by a third independent researcher mentored by the project leaders to produce a final model like the one presented here. Note: comparison of 5' and 3' UTR sequence information is not included in this GEP CURE protocol.

## Extended Data

Description: A GFF, FASTA, and PEP of DyakCAF1_Glys. Resource Type: Model. DOI: 10.22002/171sf-ny118
